# Developing a seminar curriculum for the Competence Center for General Practice in Baden-Wuerttemberg – a progress report

**DOI:** 10.3205/zma001432

**Published:** 2021-02-15

**Authors:** Sandra Stengel, Christian Förster, Monika Fuchs, Martina Bischoff, Thomas Ledig, Irmgard Streitlein-Böhme, Markus Gulich, Hannah Haumann, Jan Valentini, Anja Kohlhaas, Andreas Graf von Luckner, Dorothee Reith, Folkert Fehr, Julia Magez, Jessica Eismann-Schweimler, Joachim Szecsenyi, Stefanie Joos, Simon Schwill

**Affiliations:** 1 University Hospital Heidelberg, Department of General Practice and Health Services Research, Heidelberg, Germany; 2 University Hospital Tübingen, Institute of General Practice and Interprofessional Care, Tübingen, Germany; 3 University Hospital Ulm, Institute of General Practice, Ulm, Germany; 4 University of Freiburg, Division of General Practice, Medical Center, University Freiburg, Germany; 5 Ruhr-University Bochum, Medical Faculty, Department of General Practice, Bochum, Germany; 6 Group Practice Dr. Folkert Fehr & Dr. Jan Buschmann, Sinsheim, Germany

**Keywords:** postgraduate medical education, curriculum development, seminar curriculum, general practice, competence centers for postgraduate medical education

## Abstract

**Aim: **The seminar program of the KWBW Verbundweiterbildungplus® is offered by the Competence Center for Postgraduate Medical Education in Baden-Württemberg (KWBW) for physicians specializing in general practice (GP trainees). Attendance is a voluntary one comprised of 48 curricular units of 45 minutes each per GP trainee. This seminar program is meant to be attended in parallel to the postgraduate medical education in clinic or practice. The intention behind this project was to develop objectives, topics and a feasible structure for a seminar curriculum while taking time and financial constraints into account.

**Method: **The Kern cycle was applied in an open, modified nominal group consensus technique in the form of an iterative process. Participating were 17 experts from the departments of general practice at the universities in Freiburg, Heidelberg, Tuebingen and Ulm, plus a pediatrician.

**Results: **The main objective was defined as empowering GP trainees to independently provide high-quality primary care, including in rural areas. A basic curriculum was defined based on relevant frameworks, such as the 2018 Model Regulation for Postgraduate Medical Training (Musterweiterbildungsordnung/MWBO) and the Competency-based Curriculum General Practice (KCA). Overall, the seminar curriculum has 62 basic modules with 2 curricular units each (e.g. Basic Principles of General Practice, Chest Pain, Billing) and another 58 two-unit modules on variable topics (e.g. digitalization, travel medicine) adding up to 240 (124+116) curricular units. A blueprint with a rotation schedule for all of the teaching sites in Baden-Württemberg allows regular attendance by n=400 GP trainees over a period of five years, with individual variability in terms of program length.

**Conclusion: **The model entails a five-year, flexible program to accompany the postgraduate medical education in general practice which can also be implemented in multicenter programs and those with high enrollments. The model’s focus is on acquisition of core competencies for general practice. Despite the current shift to eLearning seminars due to SARS-CoV, the program’s implementation is being continued, constantly evaluated, and used to further develop the KWBW Verbundweiterbildung^plus^® program.

## Zusammenfassung

**Zielsetzung: **Das Seminarprogramm der KWBW Verbundweiterbildung^plus^® (Kompetenzzentrum Weiterbildung Baden-Württemberg) für Ärzt*innen in Weiterbildung Allgemeinmedizin (ÄiW AM) beinhaltet jährlich weiterbildungsbegleitend und auf freiwilliger Basis 48 Unterrichtseinheiten (UE) à 45 Min pro AiW AM. Intention des Projekts war, unter Berücksichtigung der zeitlichen und finanziellen Limitationen Ziele, Themen und eine umsetzbare Struktur eines Seminarcurriculums zu entwickeln.

**Methodik: **Der Kern-Zyklus wurde in einem offenen, modifizierten nominalen Gruppenkonsensusverfahren in einem iterativen Prozess angewendet. Insgesamt waren 17 Expert*innen aus den allgemeinmedizinischen universitären Einrichtungen Freiburg, Heidelberg, Tübingen und Ulm sowie ein Pädiater beteiligt.

**Ergebnisse: **Als Hauptziel wurde definiert, ÄiW AM zu befähigen, allgemeinmedizinisch selbständig und in hoher Qualität, auch in ländlichen Gebieten, tätig zu sein. Auf Basis relevanter Rahmenstrukturen wie der Musterweiterbildungsordnung 2018 und dem Kompetenzbasierten Curriculum Allgemeinmedizin wurde ein Basiscurriculum definiert. Insgesamt umfasst das Seminarcurriculum 62 Basismodule à 2 UE (z.B. Grundprinzipien der AM, Brustschmerz, Abrechnung) sowie weitere 58 offene Module à 2 UE (z.B. Digitalisierung, Reisemedizin) mit 240 (124+116) UE. Ein Blueprint mit Rotationsplan für alle Standorte in Baden-Württemberg ermöglicht n=400 ÄiW AM eine regelmäßige Teilnahme über 5 Jahre, die bzgl. der Dauer variiert werden kann.

**Schlussfolgerung: **Das Modell beschreibt ein 5-jähriges, flexibles, weiterbildungsbegleitendes Seminarprogramm für ÄiW AM, welches auch in multizentrischen und teilnehmerstarken Programmen umsetzbar ist und auf den Erwerb von Kernkompetenzen für die spätere allgemeinmedizinische Tätigkeit ausgerichtet ist. In der aktuellen Umstellung auf e-Learning-Seminare aufgrund von SARS-CoV wird die Implementierung fortgesetzt, kontinuierlich evaluiert und für die Weiterentwicklung der KWBW Verbundweiterbildung^plus^ genutzt.

## 1. Introduction

The Verbundweiterbildungplus postgraduate training program in Baden-Wuerttemberg was developed in 2009 in response to the shortage of general practitioners and the lack of European best-practice standards seen by international experts in the German postgraduate medical training of general practitioners [1].The program’s main objective is to ensure primary care in the future [2]. In addition to the creation of postgraduate education networks, a mentoring program, and the train-the-trainer seminars, a core component is the seminar program for physicians pursuing postgraduate medical training in general practice (GP trainees) [3]. Under the act to promote the provision of healthcare in the statutory health insurance program (GVK-Versorgungsstärkungsgesetz) [4], fifteen competence centers in Germany are now funded by Section 75a of the German Social Code with the goal of ensuring primary care in Germany [https://www.ge-weiterbildung.de/de/kompetenzzentren-weiterbildung.php]. As of 2017 in Baden-Wuerttemberg, the German Hospital Federation (Krankenhausgesellschaft), the Baden-Wuerttemberg State Chamber of Physicians (Landesärztekammer), the Baden-Wuerttemberg Association of Statutory Health Insurance Physicians (Kassenärztliche Vereinigung Baden-Württemberg), the coordinating office for general practice and the departments of general practice at the universities in Freiburg, Heidelberg, Tuebingen and Ulm have joined together to form the Competence Center for Postgraduate Medical Education in Baden-Wuerttemberg (KWBW). Together as the successor of the Verbundweiterbildungplus Baden-Wuerttemberg, they now offer the KWBW Verbundweiterbildung^plus^® postgraduate training program. The objective of the KWBW Verbundweiterbildung^plus^® program is to enhance the quality and efficiency of postgraduate medical training in general practice [5]. The participating university institutions are responsible for the postgraduate seminar program and its mandatory ongoing evaluation and quality assurance. The many years of experience gathered by the Verbundweiterbildung^plus^ can be drawn upon in the process of doing this [2], [3].

### 1.1. Starting point

**Figure 1:**
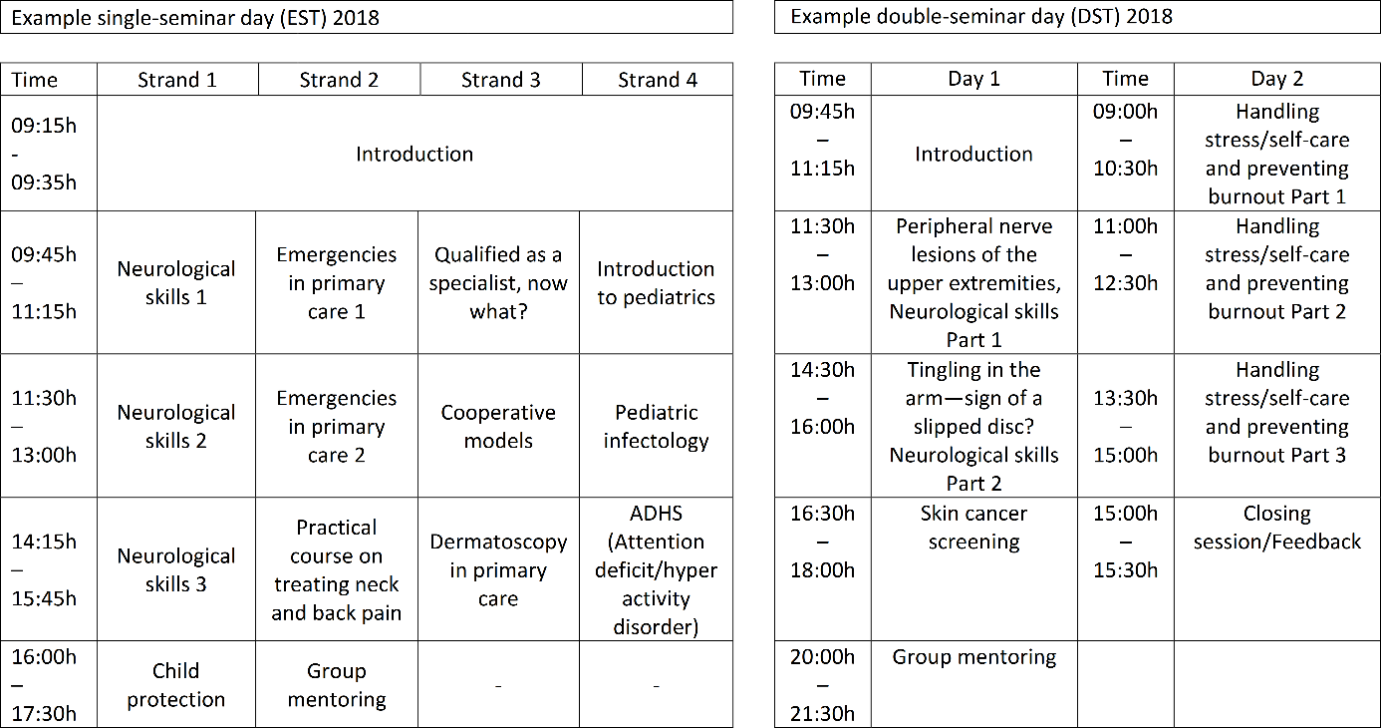
Schedules for a single-seminar day and double-seminar day at the Competence Center for Postgraduate Medical Education in Baden-Württemberg (example)

The KWBW Verbundweiterbildungplus® postgraduate training program is voluntary for GP trainees and spans all five years of the postgraduate program; it currently has 379 active participants (active = participation in the seminar program by attending at least eight course sessions within the last 12 months; as of 22/01/2020). The structure of the seminar program is outlined in figure 1 (Fig. 1). The program offers each GP trainee one double-seminar day and four single-seminar days per calendar year and encompasses a total of 48 curricular units each year. Starting on July 1, 2017, as a competence center for postgraduate medical education under Section 75a of the German Social Code, the KWBW Verbundweiterbildung^plus^® postgraduate training program has received support from public funding and from cooperative partners in the ambulatory and hospital settings [6]. It has a budget of approximately 750 Euros per GP trainee and calendar year.

On the single-seminar days that take place once per quarter at four locations in Baden-Wuerttemberg, the GP trainees can choose topics from up to four different parallel strands. The double-seminar days are held up to 14 times as two-day events with several sessions at different locations in Baden-Wuerttemberg. The seminar program has aligned itself in an unsystematic manner with the topics in the German College of General Practitioners and Family Physicians’ (DEGAM) Competency-based Curriculum General Practice (KCA) [7], [8]. A total of 185 different topics were identified in two overviews of the seminars offered between 2009 and 2016 [1], [3]. Since the program’s start there has been ongoing evaluation of the seminars using a questionnaire with a six-point Likert scale to rate content, presentation, opportunities for participation, workplace atmosphere, and relevance to practice, supplemented by the option to suggest topics and improvements and give praise [3]. The overall evaluation of the program has been rated on average as being “good” to “very good,” and the exchange of information among professional colleagues as “very good” (Likert scale 1-6; 1=very good, 6=inadequate).

#### 1.2. Purpose

At a conference for all academic KWBW Verbundweiterbildung^plus^® staff in February 2018, the desire was expressed for a curricular structure with basic topics and variable topics (see table 1 (Tab. 1), working group no. 1). This would lead to a transparent structure for the target group, comparability between the courses offered at the different university locations, easier acquisition of teachers, and improved quality assurance. The intention behind the project was to identify main objectives and develop topics and a curricular structure that are feasible, despite limited resources in terms of time, while focusing on the reliable acquisition of core competencies in general practice.

## 2. Project description

The six-step approach according to Kern (Kern cycle) [9], which is used as a common method for curriculum development in the literature [10], [11], [12], was applied here for curriculum development. These six steps encompass problem identification, general needs assessment, targeted needs assessment, definition of goals and objectives, educational strategies, implementation, evaluation and feedback. The cycle is repeated and generally worked through in a stepwise manner from step 1 to step 6. It is possible to begin with any of the six steps; switching to another step and parallel processes are possible at any time. Due to the diverse developments within Germany in recent years concerning postgraduate medical training in general practice, this process pragmatically focused on continuing the development of existing structures in Germany.

**Figure 2:**
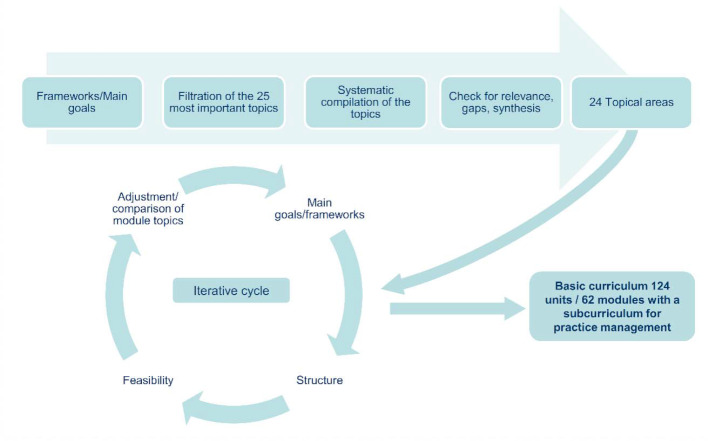
Development of a basic curriculum for general practice (124 units) as part of the entire curriculum (240 units) at the Competence Center for Postgraduate Medical Education in Baden-Württemberg

The development was carried out in a modified nominal group consensus technique [13], [14] with multicenter working groups and consensus groups that participated in an iterative process. Working groups were responsible for formulating substeps and bringing them together. Participating in this were 17 experts from the departments of general practice at the universities in Freiburg, Heidelberg, Tuebingen and Ulm, plus a pediatric specialist. Due to the close cooperation of the KWBW Verbundweiterbildungplus with medical colleagues with teaching experience who also practice in the pediatric outpatient sector, this expertise was integrated in the number of pediatric topics in the basic curriculum and the focus on basic care in ambulatory pediatrics. The composition of the consensus and working groups changed more than once over the course of developing the curriculum due to personnel turnover. The highest degree of continuity possible was ensured through an intensive exchange of information between the participants and the uninterrupted coordination. This process is presented in table 1 (Tab. 1) and figure 2 (Fig. 2).

As outlined by the Kern cycle, problem identification, general and targeted needs assessment, and the definition of main goals and objectives were undertaken first. The key piece of the work – developing a basic curriculum – was carried out in several steps. Expert groups from four universities in Baden-Wuerttemberg generated three proposals, each with 25 topics. An expert panel was held with cross-university, small-group work that yielded 24 subject areas, supplemented by brainstorming for content and an estimation of the necessary curricular units. The next step involved further developing the previously identified subject areas to fit into the topic of practice management. This main topic covers all of the selected competencies of the CanMEDS roles in the KCA. First, previously existing topics in practice management were identified in different programs (KWBW Verbundweiterbildung^plus^® seminar program, continuing education offered by the Management Akademie der Kassenärztlichen Vereinigung Baden-Württemberg (MAK), seminars held by the *Werkzeugkasten Niederlassung* of the Deutscher Hausärzteverband Baden-Württemberg). These topics were then meaningfully supplemented and developed into topics for the seminar program. Collaboration took place with the Baden-Wuerttemberg Association of Statutory Health Insurance Physicians which had already been involved in the KWBW Verbundweiterbildung^plus^® seminar program. In a next step, pediatric topics were defined more specifically. The module topics were adapted and compared in an iterative cycle until a feasible structure emerged which took implementation along with the overarching goals and frameworks into account (see table 1 (Tab. 1) and figure 2 (Fig. 2)).

## 3. Results

### 3.1. Problem identification/general needs assessment

#### 3.1.1. Relevant frameworks

The following frameworks were identified as relevant to developing the seminar curriculum with the goal of attaining core competencies in general practice:

- The Agreement on Promoting Specialty Training (Vereinbarung zur Förderung der Weiterbildung) in accordance with Section 75a of the German Social Code–Annex IV, which defines the legal framework for the competence centers and thus the conditions for funding. Among other things, it states that competence centers for postgraduate medical training should act on the basis of the existing Specialty Training Regulations/ Guideline Regulations on Specialty Training (MWBO) regarding current research in medical education and taking into consideration competency-based standards. In particular, these include the CanMEDS model (Royal College of Physicians and Surgeons of Canada); the Competency-based Curriculum General Practice (DEGAM); the model of the six core competences for medical educators (Gesellschaft für medizinische Ausbildung – GMA) [5].
- The 2018 MWBO of the German Medical Association, which is competency-based [15], and serves as guidance on the newly enacted Specialty Training Regulations for the Baden-Wuerttemberg Board of Physicians.
- The Competency-based Curriculum General Practice (KCA) DEGAM) [7], [8].
- The criteria put forth by DEGAM regarding the focus of accompanying seminars [16], [17].
- The position paper published by the Junge Allgemeinmedizin Deutschland [18].
- The frequency of primary care visits based on the data of the CONTENT project [19]. These take up a large portion of routine work in primary care medical practices and are therefore highly relevant.
- The DEGAM guidelines and others issued with DEGAM participation.
- The needs of the target group in Germany (see 3.1.3).

##### 3.1.2. Seminar curricula for GP trainees in Germany

Topic selection poses a challenge for those designing seminar programs because of the broad scope of general practice as a field. Since 2010 several authors have compiled lists of topics for seminars. In 2010, Bernau et al. developed 143 topics based on, among other sources of information, the common reasons for seeking medical care and the Dutch NIVEL network, which were then pared down to 50 topics [20]. Using a multistep process in 2009/10 and taking several international curricula into consideration, Schumann et al. developed several subject areas into a basis for developing seminars [21]. In the DEGAM working group on seminar programs in 2017, Sommer et al. compiled 884 topics from existing seminar programs at departments of general practice at German universities and used these in a multistep process to generate a proposal for 160 curricular hours entailing 111 individual topics [22]. Given the current frameworks listed above and the time constraints on the seminar program, the authors found that none of these curricula were suitable for direct transfer to the KWBW Verbundweiterbildung^plus^® curriculum.

##### 3.1.3. Needs of the target group in Germany

A literature search was performed regarding the need for postgraduate medical training among GP trainees in Germany. In Roos et al., learning about “business aspects” and “interprofessional collaboration in local care networks” were rated, at the least, as being “important” [23]. Topics on “medical expertise,” “competencies/skills,” and “reflection” were often desired [24]. Common procedures were often described as not having been satisfactorily mastered [25]. Valentini et al. show a need among GP trainees for complementary and integrative medicine as part of the specialist training in general practice [26]. A study on the risk of burnout in GP trainees showed an increased risk in comparison to an age-appropriate control group, which is why the promotion of physical and mental health is recommended in postgraduate curricula for general practice [27].

In the Verbundweiterbildungplus program, GP trainees showed a preference for “medical” topics such as dermatology or pediatrics by selecting them with a higher priority when registering for the seminar days [3]. In contrast, it is noticeable that in the final evaluations of the seminar days, the GP trainees expressed a particular wish for organizational topics such as billing or business basics [3]. It is possible to conclude that the participants are aware of their shortcomings but still do not prioritize the modules that could remedy them. For example, even in the fifth year of the postgraduate training, participants prefer to select the topic “diabetes mellitus” for the fifth time instead of one on how to run a medical practice. A curriculum could provide encouragement and incentives to eliminate this incongruence.

#### 3.2. Targeted needs assessment

The structure of the previous seminar days held by the Verbundweiterbildungplus/ KWBW Verbundweiterbildung^plus^® program are presented in figure 1 (Fig. 1). It should be noted that the target group is very heterogeneous regarding level of postgraduate training and prior knowledge. The seminar program can be started at different time points in the postgraduate GP training program, ranging from the start of the program to the fourth or fifth year of postgraduate training.

In the 2013-2018 evaluations of the Verbundweiterbildung^plus^/KWBW Verbundweiterbildung^plus^® seminar program, GP trainees expressed very diverse needs, as was expected, in response to questions about topics from all areas of the KCA (see attachment 1 ). Mentioned especially frequently (=40x) were orthopedics (n=47), wound care/chronic wounds (n=44), dermatology (n=70), depression/psyche (n=52), and Billing (n=54).

#### 3.3. Objectives

The objectives of the seminar program were defined for staff, teachers, and attendees (see attachment 2 ). The central focus of the postgraduate medical training in general practice is on the practical and supervised medical care of patients in ambulant and hospital settings. In supplement to this, the main objective of the KWBW Verbundweiterbildung^plus^® seminar program is to enable GP trainees to independently provide medical care of a high quality, including in rural and structurally weak regions. Other main objectives include forming links to practical postgraduate medical training, professional networking, peer-to-peer learning, and sustained motivation and ability to engage in life-long learning. During the project, it became increasingly clear that, in addition to defining goals, transparency concerning the program’s limitations is also explicitly important. It was stated that the seminar program could not extensively cover all of the topics in general practice and that there can be no expectation of such complete coverage. Recognizing one’s own learning needs and the motivation to take advantage of postgraduate training programs should be encouraged.

#### 3.4. Educational strategies

##### 3.4.1. Module topics for the basic curriculum with a subcurriculum on practice management

The key component of the work is identifying the 62 module topics (124 curricular units à 45 min.), which are presented in attachment 3 . They represent topics from all of the categories under “medical expertise” and the CanMEDS roles in the KCA. The spectrum ranges from the basic principles of general practice, palliative medicine, and pediatrics, to the reasons for seeking medical care, such as dyspnea, coughing, back pain, and finally to practice management topics that are made visible in a subcurriculum. Belonging to these are billing, medication and remedies, and quality management. Modules focusing on the CanMEDS roles, professionalism, physician health, and communication skills are also integrated. Relevant procedures are taught in the modules in connection with the reasons for seeking medical care (e.g. physical maneuvers in connection with vertigo, bandages in connection with injuries). The module topics can also be classified according to the competency areas of the new MWBO [15]. Generally, the relevant CanMEDS roles and procedures also need to be taken into account in terms of the content and teaching of the module (definition of learning objectives).

##### 3.4.2. Structure

**Figure 3:**
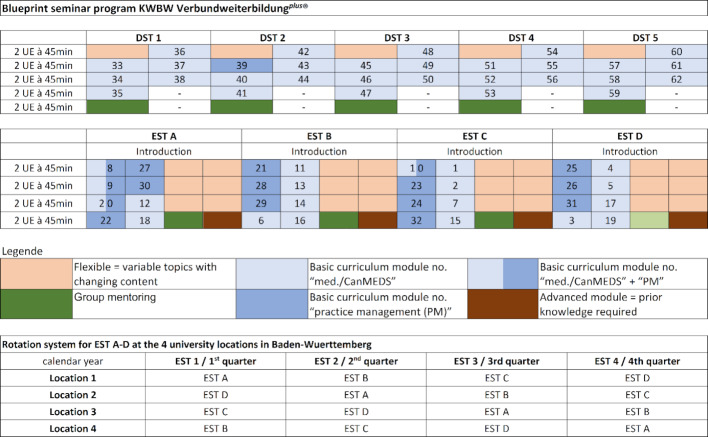
Blueprint for the KWBW Verbundweiterbildung^plus^® seminar program with rotation schedule for the individual training sites (A-D). EST = single-seminar day. DST = double-seminar day. Med. = medical. PM = practice management.

**Figure 4:**
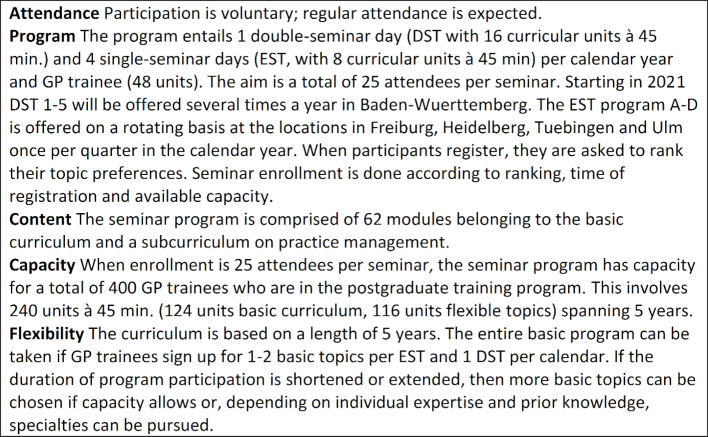
Overview of the KWBW Verbundweiterbildung^plus^® seminar program

Figure 3 (Fig. 3) presents a blueprint of the seminar program’s structure with a rotation schedule. The concept entails a five-year curriculum with flexibility for 400 GP trainees. It consists of 240 curricular units of 45 minutes each. The color coding makes it easier for the GP trainees to discern the different focal areas when registering. The basic curriculum has 62 modules (124 curricular units à 45 min.) with a visible subcurriculum on practice management. Room for flexible (=variable) topics (116 curricular units à 45 min.) enables quick response to include current topics and issues in general practice, GP trainees’ desires, and relevant topics which are not represented in the basic curriculum. For instance, topics such as digitalization, renal disease, speech therapy, and travel medicine can be covered in this way (see figure 3 (Fig. 3) und figure 4 (Fig. 4)).

#### 3.5. Implementation

Implementation of the curriculum began in January 2020 following the first announcement in the summer of 2019 and the introduction of the blueprint at the seminar days held by the KWBW Verbundweiterbildung^plus^® in November 2019. The structure was applied to the organization of the program and the blueprint began to be implemented on the seminar days held in January and February 2020. The implementation of the structure should be completed in 2020; the complete curriculum should be fully developed in 2021.

## 4. Discussion

The basic KWBW Verbundweiterbildung^plus^® curriculum entails the development of 62 modules (124 curricular units à 45 min.) for a seminar program for GP trainees which, in comparison to the curricula described above [20], [21], [22], is aligned with the current competency-based frameworks and thus focused on the acquisition of core competencies in general practice. As a way to counteract the time constraints, combining the basic curriculum with flexible (=variable) topics will allow 400 participants in Baden-Wuerttemberg to attend over five years (240 curricular units à 45 min.) with some flexibility in terms of time. The definition of main objectives and limitations serves as a guide for the GP trainees, seminar moderators and organizers. For the target group the basic curriculum provides a transparent and reliable structure that is based on the central focus of the postgraduate medical training in general practice [7], [15]. Self-directed learning is encouraged by the freedom to choose [28]; at the same time, there are incentives to participate in the topics of the basic curriculum as a result of its transparent structure. The integration of DEGAM’s and other national healthcare guidelines in the choice of topics contributes to the implementation of these guidelines [29]. With the subcurriculum on practice management, a transparent basic program is offered for GP trainees that is business-oriented and focused on the needs of the GP trainees and the requirements of the relevant frameworks. At the same time, networking is encouraged and promoted with other stakeholders in healthcare (Association of Statutory Health Insurance Physicians, professional associations of general practitioners, etc.). The introduction of a basic curriculum makes it possible to offer advanced modules as an additional category for which previously acquired knowledge is required. Thinking ahead, this can function like an incentivized system to increase motivation and deepen expertise within the seminar program.

A transfer to other competence centers in Germany is generally possible without adaptations if the number of seminar days per GP trainee and calendar year do not fall short. In Baden-Wuerttemberg each GP trainee is offered a total of six seminar days; division into single-seminar and double-seminar days is not necessary per se (but desirable for the professional exchange between the GP trainees). Furthermore, a sufficient number of attendees is needed to enable freedom of choice among the topics and for a regular (and repeated) offering of individual topics. If there is a low number of attendees and/or a low number of curricular units being offered, then an adjustment regarding the number of basic modules and/or a reduction of flexible topics will be necessary. In Baden-Wuerttemberg a total of four university institutions in general practice are working together to develop and implement the seminar topics. This work is made possible by the structure of the KWBW which allows the different locations to operate cooperatively, autonomously and equally in accordance with jointly determined standards for all of Baden-Wuerttemberg.

### 4.1. Strengths and weaknesses

The curriculum’s structure makes it possible to offer comparable courses at the four participating universities in Baden-Wuerttemberg. As a result, the design of the modules’ content can be divided up among the staff and seminar moderators at the different universities in a resource-conserving manner while still ensuring that the content is up to date with medical science and the educational sciences, as is desired and stipulated in Annex IV of Section §75a of the German Social Code V. As a consequence of this reliable structure, which is based on repetition of the basic curriculum’s topics, ongoing development of the educational strategies for teaching the content and the seminar design is possible with the inclusion of the seminar moderators in the form of quality assurance and improvement within the curriculum. A major advantage of the curriculum is that the different program lengths (extension resulting from part-time work, parental leave, shortening due to a later start in the program, etc.) or even missing individual seminar days (e.g. due to illness, inability to leave work, family responsibilities, etc.) can be responded to with flexibility.

One limitation of the consensus finding process is a result of extenuating circumstances that limited the number of working group members in working group no. 2 (see table 1 (Tab. 1)). However, it is noticeable that the agreement on the proposed suggestions was very high. Consensus group no. 3 functioned as a countervailing corrective with its high number of experts. As a result of the pragmatic multistep filtering and work process, and given the time constraints on the seminar program, the broad field of general practice simply by its nature left relevant topics uncovered. This limitation was transparently stated in the defined goals and limitations, and solutions for addressing it were proposed. Likewise, as the need to revise the KCA at regular intervals has been repeatedly pointed out [30], the prioritization of the topics should also be checked and adjusted if necessary, e.g. following a new version of the KCA. For example, the topic “megatrends” with a focus on eHealth and digitalization is not presently included in the basic curriculum, but is regularly represented in our program as a flexible topic and in podium discussions. A possible reason for not being included in the basic curriculum could be that these topics are not listed as competence objectives in the current version of the KCA.

A large percentage of the GP trainees do not participate in the KWBW Verbundweiterbildung^plus^® seminar program from the beginning of their postgraduate training. About 10% are career changers who have already qualified as medical specialists in a patient-centered discipline and thus only need to undergo a shorter period of training in general practice [31]. The question arises as to what would be more expedient: a two-year, three-year, or four-year curriculum? We explicitly chose the forward-looking five-year flexible variant with the aim of seeing participation over the entire course of postgraduate training in general practice. The advantages of participating from early on and the disadvantages of a shortened length of participation are made visible by doing this. If it were otherwise, latecomers would be motivated by the repetitions of seminars, which would conversely negatively affect those who participate in the entire program, a situation that would contradict the stated goals and objectives.

#### 4.1.1. Next steps

As an additional element of the fourth step in the Kern cycle, the definition of learning objectives and elaboration of teaching strategies was undertaken for the modules which were already being held. In the course of doing this, the pillars mentioned in Annex IV to Section 75a of the German Social Code V [6], such as the 2018 MWBO [16] , KCA with the CanMEDS roles, the GMA model of the six core competencies for medical educators [32], and competency-based education were to be taken into account. In doing so, it must be noted that important steps toward competency-based postgraduate medical training in Germany are only in the developmental phase [33], as is the introduction of the eCatalogue (eLogbuch) with the pending implementation of the 2018 MWBO [15]. Thus, the need remains for an iterative process that enables adjustment to further developments. Within the scope of this, the inclusion of megatrends and digitalization as topics in the basic curriculum should be discussed. Implementation as the fifth step in the Kern cycle is planned to take place in stages; a complete implementation should be achieved by 2021. Implementation has been delayed by the challenges of the SARS-CoV-2 pandemic necessitating a switch to eLearning seminars as of April 2020 and a resulting reduction in the program courses. At the same time, the use of the very rapidly implemented blended learning in the KWBW Verbundweiterbildungplus program is being evaluated. The question of how much space digital teaching will permanently take up in the program must be left open for now. In general, we currently view the possibility of an eLearning program and the expertise acquired from it as an enrichment, even if it is unable to completely replace the basic character of classroom teaching.

The sixth step of evaluation and feedback will take place multidimensionally. First, an evaluation by participating GP trainees will be done internally at the Competence Center. A national evaluation across all competence centers will be carried out also.

## 5. Conclusion

The KWBW Verbundweiterbildung^plus^® seminar curriculum entails a model for a five-year seminar program meant to accompany the postgraduate training in general practice offered to GP trainees by the competence centers for postgraduate medical education. This seminar program is focused on the teaching of core competencies for future medical practice as a general practitioner and allows for flexible adjustment in terms of program length. It has been possible to develop an example seminar curriculum consisting of six training days each year per GP trainee for use in multicenter and heavily attended postgraduate training programs.

## Funding

The KWBW Verbundweiterbildung^plus^® is supported by public funding under Section 75a of the German Social Code V, Annex IV. Curriculum development was based on the regulations cited here and was otherwise carried out independently.

## Acknowledgements

We wish to thank all of the seminar moderators, trainers, participants and partners of the KWBW Verbundweiterbildung^plus^®, whose dedication, joy in teaching and learning, and willingness to continue development buoys up the program. We also express our gratitude to all our cooperative partners from the ambulant and hospital settings for their valuable assistance.

## Competing interests

All of the authors except for Dr. Folkert Fehr work at the KWBW Verbundweiterbildung^plus^®. Dr. Folkert Fehr states that he works in cooperation with the KWBW. The authors declare that they have no competing interests.

## Supplementary Material

The desired topics mentioned in the written responses on the GP trainee evaluations of the Verbundweiterbildungplus /KWBW Verbundweiterbildungplus® (January 2013 – July 2018)

Goals and limitations of the KWBW Verbundweiterbildungplus® seminar program

KWBW Verbundweiterbildungplus® basic curriculum with a sub-curriculum in practice management as a component of the overall curriculum (240 curricular units)

## Figures and Tables

**Table 1:**
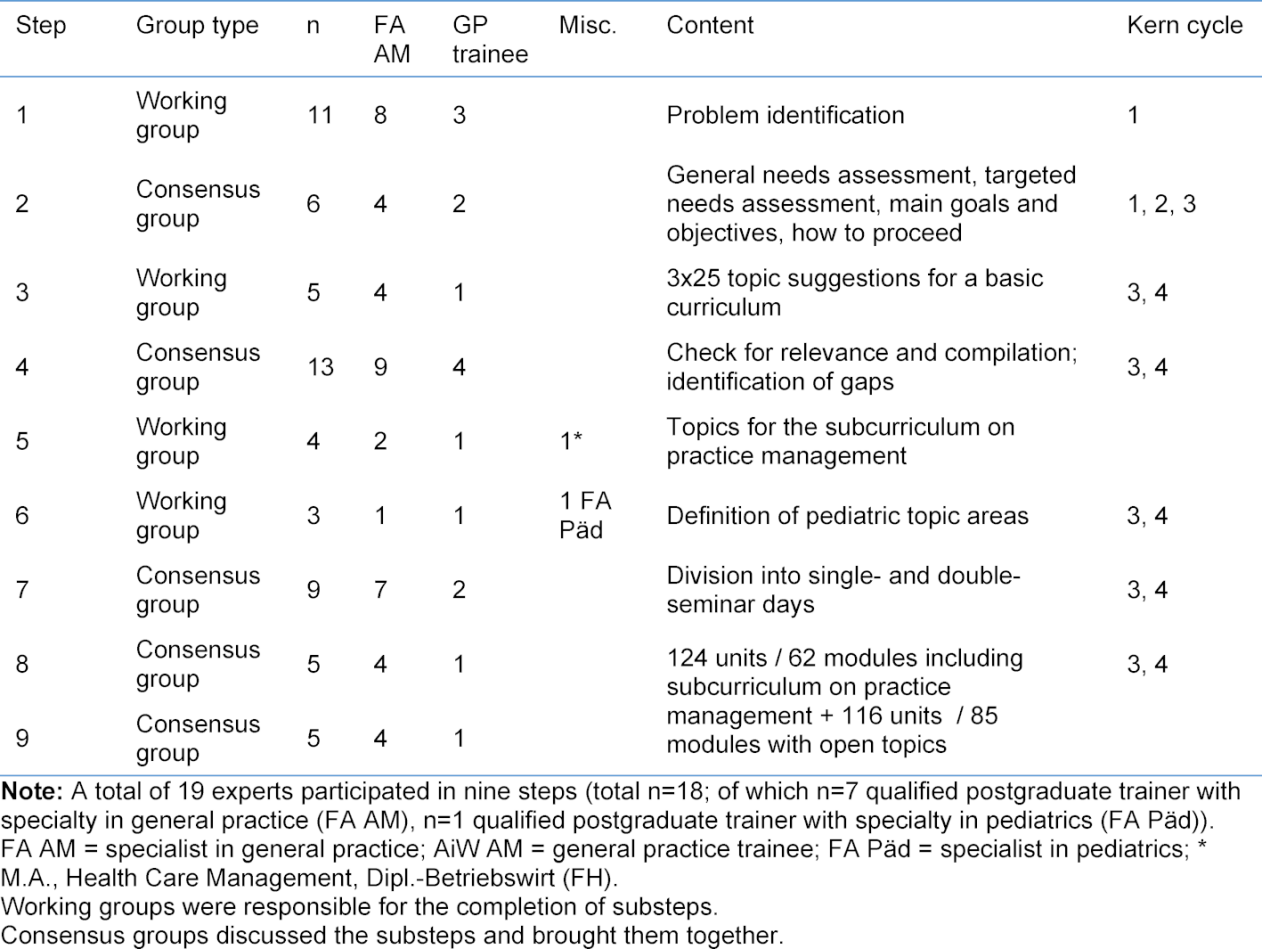
Participating experts
