# Supplementary material for: Developing a seminar curriculum for the Competence Center for General Practice in Baden-Wuerttemberg – a progress report
Source: GMS J Med Educ. 2021 Feb 15;38(2):Doc36. doi: 10.3205/zma001432 (PMC7958914; doi:10.3205/zma001432)
Supplement: The desired topics mentioned in the written responses on the GP trainee evaluations of the Verbundweiterbildungplus /KWBW Verbundweiterbildungplus® (January 2013 – July 2018) [file JME-38-2-36-s-001.pdf]

Attachment 1: The desired topics mentioned in the written responses on the GP trainee evaluations of the Verbundweiterbildung*plus* /KWBW Verbundweiterbildung*plus®* (January 2013 – July 2018)

**Category: "Competency-based Curriculum General Practice"**	**n (total)**	**Topics**	**n**
Metabolism, Nutrition, Digestive system	64		
		Diabetes mellitus	34
Cardiovascular system	25		
Respiratory organs and ear	39		
		ENT	20
Musculoskeletal system, Pain, Injuries	136		
		Orthopedics	47
		Back pain	27
		Wound care/Chronic wounds	44
Eye and nervous system	43		
		Headaches	10
		Neurology	15
		Vertigo	12
Sexuality, Family planning, Sexual organs, Urinary tract	32		
		Gynecology	15
Skin	83		
		Dermatology	70
		Skin cancer screening	13
Changes in consciousness, Cognition, Emotion	62		
		Addiction	10
		Depression/Psyche	52
Special aspects of children and adolescents	59		
		Pediatrics	28
		Immunizations	31
Caring for chronically ill and elderly patients	92		
		DMP (Disease Management Program)	14
		Chronic pain	13
		The elderly patient	20
		Polypharmacy/Medication monitoring	28
Palliative medicine	20		
		Palliative medicine	15
Emergencies	34		
		Emergencies	22
		Pediatric emergencies	5
Communication	23		

Attachment 1 to: Stengel S, Förster C, Fuchs M, Bischoff M, Ledig T, Streitlein-Böhme I, Gulich M, Haumann H, Valentini J, Kohlhaas A, Graf von Luckner A, Reith D, Fehr F, Magez J, Eismann-Schwemmler J, Szecsenyi J, Joos S, Schwill S. Developing a seminar curriculum fort he Competence Center for General Practice in Baden-Wuerttemberg – a progress report. GMS J Med Educ. 2021;38(2):Doc36. DOI: 10.3205/zma001432

		Communication skills training	10
		Communication with difficult patients	12
**Category: "Competency-based Curriculum General Practice"**	**n (total)**	**Topics**	**n**
Collaboration	12		
Management	134		
		Billing	54
		Now a specialist/taking up practice	23
		Practice organization	34
		Practice software	10
Representing the patient: Guidance through the healthcare system and health promotion	72		
		Aids	11
		Social medicine	28
		Medications and remedies	19
Learning and teaching	21		
		How do I structure my postgraduate training?	11
Professionalism	38		
		Law	17
Procedures	135		
		ECG (electrocardiogram)	10
		Minor surgery	10
		Complementary medicine	15
		Orthopedic exam	25
		Sonography	17
		Routine infant/adolescent checkups	15
Miscellaneous	120		
		Antibiotics	11
		Caring for asylum seekers	10
		Nutritional medicine	16
		Manual therapy	10
		Preparation for specialist consultation	13
		Additional post-licensure training	16

Note: n= number of the desired topics mentioned. Listed are all topics which had been mentioned a minimum of 10 x during the time period.

Method: The topics desired by the attendees from the evaluation questionnaires from 2013 to 2018 in the Verbundweiterbildung*plus*/KWBW Verbundweiterbildung*plus®* postgraduate training program were categorized, assigned to the subject areas of the DEGAM Competency-based Curriculum General Practice, and the frequency of the mentions was calculated.

Attachment 1 to: Stengel S, Förster C, Fuchs M, Bischoff M, Ledig T, Streitlein-Böhme I, Gulich M, Haumann H, Valentini J, Kohlhaas A, Graf von Luckner A, Reith D, Fehr F, Magez J, Eismann-Schwemmler J, Szecsenyi J, Joos S, Schwill S. Developing a seminar curriculum fort he Competence Center for General Practice in Baden-Wuerttemberg – a progress report. GMS J Med Educ. 2021;38(2):Doc36. DOI: 10.3205/zma001432
