# Supplementary material for: Developing a seminar curriculum for the Competence Center for General Practice in Baden-Wuerttemberg – a progress report
Source: GMS J Med Educ. 2021 Feb 15;38(2):Doc36. doi: 10.3205/zma001432 (PMC7958914; doi:10.3205/zma001432)
Supplement: Goals and limitations of the KWBW Verbundweiterbildungplus® seminar program [file JME-38-2-36-s-002.pdf]

Attachment 2: Goals and limitations of the KWBW Verbundweiterbildung$^{plus®}$ seminar program

The central focus of the postgraduate program in general practice is on the practical and supervised medical care of patients in the ambulatory and hospital settings.

Main objective
The main objective of the KWBW Verbundweiterbildung$^{plus®}$ seminar program is to enable General Practice (GP) trainees to independently provide high-quality primary care, including in rural and structurally weak regions.

Main goals of the activities offered in parallel to the postgraduate medical training program

- Building and supporting a professional network (peer-to-peer, focused on general practice, interdisciplinary, interprofessional, Medical Association, professional medical associations, KV, DEGAM, JADE, university departments of general practice)
- Encouragement of professional exchange among colleagues
- Fostering an identification with GP
- Ongoing motivation and empowerment of GP trainees to actively shape their postgraduate education and training
- Mastering an open approach to handling challenging situations in postgraduate medical training and mutual support in an open, structured conversation (mentoring)
- Motivation to interact and give feedback between GP trainees and mentors
- Experiencing joy in life-long learning and "learning to learn"
- Motivation to take advantage of additional advanced training programs that are product neutral and free of pharmaceutical industry interests.

Goals and limitations of the seminar days

- The seminar days accompany the practical training in the ambulatory and hospital settings.
- The seminar days contribute to expanding and deepening practical clinical knowledge, experience, and reflection on attitudes and skills.
- The seminar days help in the acquisition of indispensible basic knowledge and skills in general practice.
- The content of the seminar days is based on the valid rules and regulations applying to postgraduate education (see the Specialty Training Regulations for Baden-Wuerttemberg, the Guideline Regulations on Specialty Training (MWBO 2018), and the Competency-based Curriculum General Practice, including the CanMEDs competencies.
- The seminar days are explicitly trainee-centered and designed to be interactive.
- The main focus is on helpful, cooperative problem solving (encouraging networking and teamwork).
- The expertise of the attendees (GP trainees) is to be included (peer-to-peer learning and teaching).
- The moderators of the KWBW Verbundweiterbildung$^{plus®}$ seminars inspire the attendees to prepare themselves professionally and encourage their cooperation.
- Not all of the GP topics can be covered in full by the seminar program and there is no claim that this is possible. The central concern, alongside the stated goals, is the recognition of one's own learning needs, the targeted planning of one's own future steps in a particular phase of postgraduate study, and the motivation to take advantage of additional advanced training programs.

The goals and objectives were formulated on the basis of Annex IV to the Agreement on Promoting Postgraduate Medical Education under Section 75a of the German Social Code V; DEGAM position paper – Criteria for competence centers for general practice.

Attachment 2 to: Stengel S, Förster C, Fuchs M, Bischoff M, Ledig T, Streitlein-Böhme I, Gulich M, Haumann H, Valentini J, Kohlhaas A, Graf von Luckner A, Reith D, Fehr F, Magez J, Eismann-Schwemmler J, Szecsenyi J, Joos S, Schwill S. Developing a seminar curriculum fort he Competence Center for General Practice in Baden-Wuerttemberg – a progress report. GMS J Med Educ. 2021;38(2):Doc36. DOI: 10.3205/zma001432
