# Supplementary material for: Developing a seminar curriculum for the Competence Center for General Practice in Baden-Wuerttemberg – a progress report
Source: GMS J Med Educ. 2021 Feb 15;38(2):Doc36. doi: 10.3205/zma001432 (PMC7958914; doi:10.3205/zma001432)
Supplement: KWBW Verbundweiterbildungplus® basic curriculum with a sub-curriculum in practice management as a component of the overall curriculum (240 curricular units) [file JME-38-2-36-s-003.pdf]

Attachment 3: KWBW Verbundweiterbildung*plus*® basic curriculum with a sub-curriculum in practice management as a component of the overall curriculum (240 curricular units)

**KWBW Verbundweiterbildung*plus*® basic curriculum 124 units – 62 modules**

**No.**	**Module topics and single-seminar days**
1	Diabetes mellitus - Part 1
2	Diabetes mellitus - Part 2
3	Colds, coughs, hoarseness – symptomatic treatment or antibiotics?
4	Dyspnea / coughing with focus on asthma with DMP[1]
5	Dyspnea / coughing with focus on COPD[2] with DMP[1]
6	Complementary medicine
7	Blind spot – e.g. sexually transmitted diseases (STD), sexual medicine
8 +PM	Back/neck pain with remedies - Part 1
9 +PM	Back/neck pain with remedies - Part 2
10 +PM	Joint pain, muscle pain (without trauma) with aids
11	Vertigo
12	Urination complaints
13	Skin - Part 1
14	Skin - Part 2
15	The acutely sick child/adolescent (who was always healthy before)
16	Long-term care of chronically ill children/adolescents
17	Psychologically troubled children/adolescents
18	Youth occupational safety checkups, preventive checkups for children and adolescents (U10-J2), including immunizations
19	Routine preventive checkups for children (U1 – U9)

20 +PM	Emergencies
21 PM	Billing
22 PM	Physician as team leader / Team
23 PM	QM[3] – Risk and error management + GDPR[4] - Part 1
24 PM	QM[3] + Risk and error management + GDPR[4] - Part 2
25 PM	Social medicine, rehabilitation, benefits/entitlements, mother-child convalescent care, incapacity-to-work certificates
26 PM	Prescriptions/pharmaceutical guidelines
27 PM	Questions about immunization
28 PM	Association of Statutory Health Insurance (SHI) Physicians 1: Doctor's office
29 PM	Association of SHI Physicians 2: Business management
30 PM	Association of SHI Physicians 3: Billing
31 PM	Association of SHI Physicians 4: Demand for consultation hours
32 PM	Association of SHI Physicians 5: Legal aspects

**No.**	**Module topics for double-seminar days (DST)**

**No.**	**DST 1**
33	Basic principles / Uncertainty
34	Inappropriate treatment, over/under treatment / evidence-based medicine
35	Headaches
36	Attitude / professionalism
37	Chest pain – focus on coronary heart disease (Part 1)
38	Chest pain –focus on coronary heart disease (Part 2) / Dyspnea – focus on heart failure

**No.**	**DST 2**

39	Care of chronically ill patients
40 PM	Organization of care / delegation
41	Acute situations / decompensation in chronically ill patients
42	Care of elderly patients
43	Polypharmacy / drug safety
44	House visit with training

**No.**	**DST 3**
45	Abdominal pain, nausea, diarrhea - Part 1
46	Abdominal pain, nausea, diarrhea - Part 2
47	Abdominal pain, nausea, diarrhea - Part 3
48	Injuries / wounds - Part 1
49	Injuries / wounds - Part 2
50	Injuries / wounds - Part 3

**No.**	**DST 4**
51	Palliative organization
52	Palliative symptoms including chronic pain therapy
53	Palliative adjuvant therapy (aromatherapy, wraps, etc.)
54	Prevention with arterial hypertension, hyperlipidemia, checkups, preventive care
55	ARRIBA / Informed shared decision making, communication skills training
56	Palliative patient medical directives / organ donation

No.	DST 5
57	Exhaustion / sleep disorders / mood swings / weakness - Part 1
58	Exhaustion / sleep disorders / mood swings / weakness - Part 2
59	Anxiety / panic

Attachment 3 to: Stengel S, Förster C, Fuchs M, Bischoff M, Ledig T, Streitlein-Böhme I, Gulich M, Haumann H, Valentini J, Kohlhaas A, Graf von Luckner A, Reith D, Fehr F, Magez J, Eismann-Schwemmler J, Szecsenyi J, Joos S, Schwill S. Developing a seminar curriculum fort he Competence Center for General Practice in Baden-Wuerttemberg – a progress report. GMS J Med Educ. 2021;38(2):Doc36. DOI: 10.3205/zma001432

60	Addiction
61	Reflection (medical professionalism, physicians' health)
62	Communication skills training

Note PM = practice management; +PM = medical/CanMEDS focus and relevant PM content; UE = curricular unit

[1]DMP = Disease Management Program; [2] COPD = Chronic Obstructive Pulmonary Disease; [3] QM = Quality Management; [4] GDPR = General Data Protection Regulation
